# Effects of the Small‐Molecule ISRIB on the Rapid and Efficient Myelination of Oligodendrocytes in Human Stem Cell‐Derived Cerebral Organoids in Patients With Leukoencephalopathy With Vanishing White Matter

**DOI:** 10.1111/cns.70398

**Published:** 2025-04-28

**Authors:** Wei Yan, Jiong Deng, Jie Zhang, Kai Gao, Huan Yi, Junjiao Zhang, Fan Zhang, Jingmin Wang, Yuwu Jiang, Ye Wu

**Affiliations:** ^1^ Children's Medical Centre Peking University First Hospital Beijing China

**Keywords:** astrocytes, ISRIB, leukoencephalopathy with vanishing white matter, oligodendrocytes, rapid myelinating oligodendrocyte brain organoids, unfolded protein response

## Abstract

**Introduction:**

Leukoencephalopathy with vanishing white matter (VWM) is a rare genetic disorder caused by mutations in any one of the *EIF2B*1–5, which encode subunits of eukaryotic translation initiation factor 2B (eIF2B). Previous studies suggested that the dysfunction of astrocytes played a central role in the pathogenic mechanism of VWM. In addition, eIF2B participates in the unfolded protein response(UPR) by coordinating the integrated stress response (ISR). Higher susceptibility to endoplasmic reticulum stress (ERS) and abnormal overactivation of the unfolded protein response (UPR) were found in VWM, which led to logical deterioration and exacerbation of cell death. There are currently no specific treatments available for VWM.

**Aim:**

Previous studies have successfully constructed three‐dimensional brain organoids that can be used to study the development of neuronal cells during brain development. In this study, we aimed to develop a more rapid and efficient brain organoid model that would produce mature astrocytes, oligodendrocytes, and myelin within 8 weeks. The small‐molecule ISR inhibitor (ISRIB) is a specific eIF2B activator by inhibiting the phosphorylation of eukaryotic translation initiation factor 2 (eIF2). Thus, ISRIB is used on eIF2B mutant organoids to determine its potential as a therapeutic approach for VWM.

**Results:**

We constructed *EIF2B4* and *EIF2B5* mutants as well as wild‐type rapid myelinating oligodendrocyte brain organoids using human induced pluripotent stem cells (iPSCs). We observed mature astrocytes, oligodendrocytes, and myelin within 8 weeks, greatly shortening the culture period. Compared with the wild type, mutant organoids displayed a smaller size and contained increased immature and dysfunctional astrocytes, oligodendrocytes, and sparse myelin. Abnormal overactivation of the UPR pathway was also present in mutant cerebral organoids. Additionally, we found that the maturation and function of these cells in mutant organoids were significantly improved after ISRIB treatment, which also inhibited hyperactivation of the unfolded protein response (UPR) signaling pathway.

**Conclusions:**

Our study established a rapid myelinating oligodendrocyte brain model in VWM for the first time, providing a more effective and tractable platform for further study of this condition and other white matter diseases. Furthermore, our findings suggested that ISRIB may have potential as a clinical treatment for VWM.

## Graphical abstract

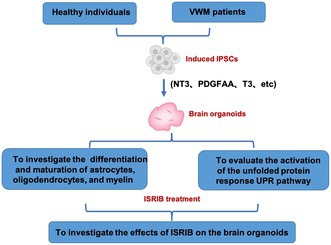

## 1 Introduction

Leukodystrophy with vanishing white matter (VWM) is a rare, autosomal recessive inherited leukodystrophy that usually occurs in childhood. It is characterized by cerebellar ataxia, spasticity, progressive motor decline, and rapid deterioration provoked by stressors such as infections, acute fright, and minor head trauma [1, 2]. The age of onset of VWM varies from early infancy or even antenatal to adulthood, while the clinical symptoms and disease severity are also highly variable. Rapid progression is generally associated with early‐onset VWM [3, 4].

Biallelic pathogenic variants in any of the housekeeping genes *EIF2B*1–5, which encode the five subunits of eukaryotic translation initiation factor 2B (eIF2B), lead to VWM by reducing eIF2B activity [5, 6]. eIF2B is a guanine nucleotide exchange factor (GEF) which is essential for mRNA translation initiation and protein synthesis. The phosphorylation of eukaryotic translation initiation factor 2 (eIF2) is considered a competitive inhibitor of eIF2B that regulates eIF2B‐GEF activity [7, 8]. In addition, eIF2 phosphorylation participates in the initiation of the integrated stress response (ISR), which is associated with several neurodegenerative disorders. Previous studies have shown that mutations in *EIF2B1–5* lead to reductions in eIF2B GEF activity, triggering improper activation and dysregulation of the ISR. Moreover, the constitutive activation of the ISR attenuates eIF2B‐GEF activity through the phosphorylation of eIF2. Unfolded proteins in the endoplasmic reticulum (ER) bind to BiP (GRP78), subsequently leading to the activation of transmembrane ER proteins, including IRE1, PERK, and ATF6, which together constitute the unfolded protein response (UPR). The integrated stress response (ISR) represents one of the three UPR pathways, where eIF2B plays a crucial role, acting at a pivotal juncture following the induction of ER stress and the activation of PERK. During periods of ER stress, IRE1 identifies and cleaves two specific stem–loop sequences within the XBP‐1 mRNA, yielding a larger protein known as spliced XBP1 (XBP1‐S). XBP1‐S functions as a critical transcription factor, positively regulating cellular proliferation and angiogenesis. Under ER stress conditions, ATF6 is cleaved to produce its active form of 50 kDa (ATF6‐N), which translocates to the nucleus to initiate transcriptional activation [9, 10]. Contemporary studies have highlighted the overactivation of the ISR and the PERK‐eIF2‐ATF4‐CHOP pathway in vanishing white matter (VWM), although the activation status of all three branches of the UPR in VWM remains a topic of debate [11, 12, 13]. Research conducted by van der Knaap has indicated that there is not only activation of the PERK pathway but that all three branches of the UPR are indeed activated in the brain tissues of patients afflicted with VWM [14]. Furthermore, an enhanced expression of UPR markers appears to be predominantly observed in oligodendrocytes and astrocytes, both of which are known to play significant roles in the pathology of VWM [14]. However, the brain samples obtained from VWM patients are typically post‐mortem and represent stages of the disease that are markedly advanced. Consequently, the observed overactivation of the unfolded protein response (UPR) may be attributed to the terminal stages of the disease process. In the context of VWM mouse models associated with the *EIF2B5* R191H mutation, which is homologous to the human R195H mutation, there is no evidence of activation of the UPR [15, 16]. Moreover, similar findings have been observed in VWM mouse models that carry mutations in either *EIF2B4* or *EIF2B1*, wherein UPR activation has not been demonstrated [13, 17]. Therefore, in this study, we aimed to investigate the expression levels of the three UPR arms—ATF4, the active form of ATF6 (ATF6‐N), and spliced XBP1 (XBP1‐S)—to ascertain whether there is indeed an activation of all three branches of the UPR in the context of VWM.

Currently, most clinical treatments for VWM are symptomatic therapies. However, considering the critical role of the ISR in the pathogenesis of VWM, drugs targeting the components of the ISR could be effective potential therapies for VWM. The small‐molecule ISR inhibitor (ISRIB) is a specific eIF2B activator that inhibits the phosphorylation of eIF2 and can cross the blood–brain barrier [13]. In mouse models, ISRIB has been confirmed to promote memory formation without causing severe toxicity [18]. Although previous studies based on cell and animal models have provided valuable evidence for the positive effects of ISRIB on VWM and other disorders characterized by abnormal ISR activation, developing a three‐dimensional brain model of VWM stem cells from affected patients could be a more accessible route for exploring its impact. In our previous work, the eIF2B‐mutant cerebral organoids were constructed by Deng et al. to explore the brain developmental process [19]. According to this method, mature astrocytes were observed at approximately week 12, while Olig2‐positive mature oligodendrocytes were detected at nearly week 20. In addition, it took more than 180 days for the maturation of myelin sheaths in normal brain organoids and even longer in cerebral organoids of VWM.

Here, we constructed a rapid myelinating oligodendrocyte (OL) brain organoids model for VWM in which mature astrocytes, oligodendrocytes, and myelin were identified within 56 days following the instructions of Mohammed R. Shaker et al. [20]. In this rapid protocol, a series of cellular growth factors that promoted the proliferation and maturation of oligodendrocytes were applied, which contributed to the acceleration of organoid development. In comparison to the methods of cerebral organoids constructed by Deng et al., the accelerated development phase induced by specific growth factors in our model might lead to predominantly differentiation and excessive enrichment of cells of the oligodendrocyte and astrocyte lineage, causing a more remarkable difference in expression levels of glial cells between the wild‐type and eIF2B‐mutant brain organoids. Therefore, this innovative method for generating human cortical brain organoids can be a more effective in vitro tool for studying VWM or other white matter disorders. For exploring the precise dynamic organoid differentiation process in most neurological disorders, the previously pioneering organoid methodology offers more distinct advantages. Furthermore, we investigated the influence of ISRIB on eIF2B mutant organoids. The results showed that ISRIB could reduce the overactivation of the UPR pathway and improve the function of astrocytes and oligodendrocytes in brain organoids, indicating that ISRIB could be a potentially efficient treatment for VWM.

## 2 Materials and Methods

### 2.1 Generation and Maintenance of Induced Pluripotent Stem Cells

We obtained iPSCs from two patients with VWM caused by mutations in *EIF2B5* and *EIF2B4*, respectively. VWM1 patient‐derived iPSCs were generated from human dermal fibroblasts (HDFs) through neon electroporation using Yamanaka factors (OCT3/4, SOX2, KLF4, and L‐MYC) according to the manufacturer's instructions for the Epi5 Episomal iPSC Reprogramming Kit (Invitrogen, A15960) [21]. Similarly, we generated VWM2 patient‐derived iPSCs. The genotype of VWM1 was *EIF2B5*: c.1827_1838del (p.610_613del4) c.1157G>A (p.Gly386Val), and the genotype of VWM2 was *EIF2B4*c.932 T>C (p.Ile311Thr) c.1195A>C (p.Lys399Gln) [19]. The first patient (VWM1) was a male who succumbed to the disease at the age of 16. His onset age was 4 and the initial symptom was motor deterioration triggered by head trauma. The VWM2 patient was a male with the onset characterized by regression of motor function and epileptic seizures at the age of 12 months. Further details on the clinical characteristics and MRI findings of these patients can be found in our previous research [19, 21]. Two human wild‐type (WT) iPSC lines were obtained from Beijing Cellapy Biotechnology Co. Ltd., and their cell qualities were determined. Written informed consent was obtained, and the study was approved by the Biomedical Ethics Committee of Peking University First Hospital. All iPSC lines were cultured on Matrigel (StemCell Technologies, Cat. #354277) in mTeSR (Stem Cell Technologies, Cat. #85851) according to the manufacturer's instructions. When the percentage of IPSC colonies reached 70%–80%, the cells were passaged using enzyme‐free Gentle Cell Dissociation Reagent (GCDR, STEMCELL Technologies, 100–0485) and incubated with mTeSR containing 10 μM ROCKi Y‐27632 (Lonza‐PeproTech, 1,293,823‐B) for 24 h.

### 2.2 Culture of Rapid Myelinating Oligodendrocyte Brain Organoids

Rapid myelinating oligodendrocyte (OL) brain organoids were generated from human iPSCs following the protocol described by Mohammed R. Shaker et al. [20]. Initially, iPSCs were plated on Matrigel in six‐well plates at a density of 20%–30%, and healthy colonies with no detectable differentiation were generated. To induce human neuroectodermal (hNEct) cells, the iPSCs were treated with the dual SMAD inhibitors SB‐431542 (10 μM) and LDN 193189 (100 nM) for 3 days in N2 medium, which consists of DMEM/F12 (Gibco, Cat. #11320–33), 2% B‐27 supplement (Gibco, Cat. # 17504044), 1% N‐2 supplement (Gibco, Cat. #17502–048), 1% MEM nonessential amino acids (Gibco, Cat. #11140–050), 1% penicillin/streptomycin (Gibco, Cat. #15140148), and 0.1% β‐mercaptoethanol (Gibco, Cat. #21985–023). Then, these cells were detached by dispase enzyme (2.4 unit/mL) for no more than 30 min at 37°C, and colonies were transferred to a 15 mL tube using a wide‐bore P1000 pipette tip, which allowed them to sink to the bottom of the tube due to the influence of gravity. These 2D neuroectodermal colonies were then generated into 3D neuroectodermal spheroids in a six‐well ultralow attachment plate for 4 days in OL differentiation medium (OLDM). OLDM consists of DMEM/F12 (Gibco, Cat. #11320–33), 2% B‐27 minus vitamin A supplement (Gibco, Cat. # 17504044), 1% N‐2 supplement (Gibco, Cat. #17502–048), 1% MEM nonessential amino acids (Gibco, Cat. #11140–050), 1% penicillin/streptomycin (Gibco, Cat. #15140148), 0.1% β‐mercaptoethanol (Gibco, Cat. #21985–023), human IGF‐I (Lonza‐PeproTech, 100–11‐100), insulin (Life Technologies, 12,585,014), human NT‐3 (PeproTech, 450–03‐50), 3,3′,5‐triiodo‐L‐thyronine (Sapphire Bioscience, 000–23,845), HGF (Lonza‐PeproTech, 100‐39H) biotin (Sigma, B4639), cAMP (Sigma, D0627), and PDGF‐AA (Lonza‐PeproTech, 100‐13A). Notably, 40 ng/mL bFGF was added to each well daily to promote the formation and proliferation of neuroepithelial spheroids. Similar‐sized neural spheroids (500 μm) were selected and transferred to parafilm‐dimples using a wide‐bore 100 μL pipette tip and were incubated at 37°C for 30 min with 20 μL of basement matrix. After incubation, the embedded spheroids were maintained in a low‐attachment six‐well plate using a P1000 tip with 4–5 mL of Olig3 medium consisting of DMEM/F12: Neurobasal (1:1) medium, 1% B27 without vitamin A, 0.5% N2, 1% GlutaMAX, 1% penicillin/streptomycin, 10 ng/mL PDGF‐AA, 10 ng/mL IGF, 10 ng/mL NT3, 60 ng/mL T3, 100 ng/mL biotin, 10 μM cAMP, 10 ng/mL HGF, 1% NEAA, 0.35 μL/mL β‐mercaptoethanol (17.5 μL in 50 mL medium), and 25 μg/mL insulin (Sigma, cat. no. I9278‐5 M: 12.5 μL in 50 mL medium). During the first week of differentiation, the medium was changed every 3 days. When the organoids grew increasingly larger, the medium was changed every 2 days.

### 2.3 Treatment With the Small‐Molecule ISRIB

The WT and mutant organoids were treated with 500 μM ISRIB (MCE or HY‐12495A) after the neuroectodermal spheroids were embedded in Matrigel for 1 week. Fresh ISRIB was added when we changed the medium to ensure that the organoids were constantly maintained with ISRIB at an adequate concentration.

### 2.4 Immunofluorescence Analysis

The organoids were first fixed in 4% paraformaldehyde (PFA) solution at 4°C overnight and then washed with 1× phosphate‐buffered saline three times for 10 min at room temperature. The fixed organoids were then equilibrated in 30% sucrose solution at 4°C overnight, embedded in OCT compound, and stored in a −80°C freezer. Subsequently, the organoid samples were sectioned into 15‐μm‐thick serial sections and treated with 5% bovine serum albumin (BSA) for 1 h. After blocking, the sections were incubated overnight at 4°C with primary antibodies specific for various proteins, including GFAP (CST, 1:300, 3670 S), PDGFRα (Santa Cruz, 1:200, ab61219), Olig2 (Abcam, 1:100, ab109186), myelin basic protein (MBP, CST, 1:100, ab62631), GFAPδ (EMD Millipore, 1:500, AB9598), nestin (CST, 1:200, 4760 S), αB‐crystallin (Abcam, 1:200, ab76467), O4 (Sigma–Aldrich, 1:100, MAB1326), ATF4 (CST, 1:100, 11,815 S), ATF6 (Abcam, 1:100, ab122897), and XBP1‐S (CST, 1:100). After washing with 1 × PBS three times for 10 min each, the labeled tissues were incubated with secondary antibodies for 1 h at room temperature. Finally, all slices were stained with DAPI and stored at 2°C–8°C. In this study, all images were captured by confocal microscopy, and ImageJ was used to quantify the area of fluorescence expression in the positive cells from the confocal images.

### 2.5 Transmission Electron Microscopy

The OL organoids were washed with PBS three times and then fixed with 2.5% glutaraldehyde at 4°C overnight. After fixation, the organoids were contrasted with 1% osmium tetroxide for 90 min, dehydrated with a series of ethanol solutions ranging from 50% to 100%, and then embedded in EPON. Subsequently, ultrathin sections (70 nm) were cut using an ultramicrotome and stained with uranyl acetate and lead citrate for 10 and 12 min, respectively. Finally, the organoid sections were observed by transmission electron microscopy. The G‐ratio, which is calculated by ImageJ software, is widely used to measure the relative thickness of myelin around an axon.

### 2.6 Quantitative Real‐Time Polymerase Chain Reaction (q‐PCR)

Total RNA was extracted from brain organoids using Trizol. The cDNA was synthesized using the Revert Aid First Strand cDNA Synthesis kit (ThermoFisher Scientific, UAB, K1622). To quantify the expression of Xbp1 spliced (Xbp1‐S) and ATF4, q‐PCR was performed using SYBR (Part No. 4368577, Applied Biosystems, UK). Primer sequences for Xbp1 spliced (XBP1‐S) and ATF4 were used as follows: XBP1‐S‐forward: 5′‐TCAGACTACGTGCGCCTCT‐3′ and XBP1‐S‐reverse: 5′‐CTCTGGGGAAGGACATTTGA‐3′; ATF4‐forward: 5′‐TGGCGCTTCTCACGGC‐3′ and ATF4‐reverse: 5′‐GTCTTTGTCGGTTACAGCAACG‐3′. The primer sequences for GAPDH were 5′‐AGGTCGGTGTGAACGGATTTG‐3′ and 5′‐GGGGTCGTTGATGGCAACA‐3′.

### 2.7 Western Blot Analysis

RIPA lysis buffer was used to prepare lysates from brain organoids. Proteins and protein ladder (10–180 kDa, Thermo Scientific, 26,616) in the samples were separated by sodium dodecyl sulfate–polyacrylamide gel electrophoresis of 8%–15% and then transferred to nitrocellulose membranes. The membranes were blocked in 5% skimmed milk (in PBS, pH 7.2, containing 0.1% Tween‐20) and the proteins on the membrane were incubated overnight at 4°C with primary antibodies for ATF6(Abcam, 1:1000), Xbp1 spliced (XBP1‐S) (CST,1:1000, #12782) and GAPDH (Santa Cruz Biotechnology, 1:1000). The secondary antibodies used were HRP‐conjugated polyclonal sheep anti‐rabbit (ZSGB‐Bio, 1:1000, ZB‐2301) or anti‐mouse IgG (ZSGB‐Bio, 1:1000, ZB‐2305). The epitope was visualized by an ECL Western blot detection kit (Millipore Corporation, Billerica, MA, USA).

### 2.8 Statistical Analysis

Statistical analysis was performed by SPSS v26.0 and GraphPad Prism v9.0 software. The data are presented as the means ± SD. *p* values < 0.05 were considered to indicate statistical significance, while ns indicates no statistical significance. All the experiments were repeated at least three times in our study. The Kolmogorov–Smirnov test was performed to check the normal distribution of the data. For two or three groups of normally distributed data, Two‐tailed *t*‐tests or one‐way ANOVA were applied. Mann–Whitney U and Kruskal–Wilcoxon tests were performed for other data. The proportion of positive cells in the confocal images was quantified using ImageJ software.

## 3 Results

### 3.1 Maturation of Astrocytes, Oligodendrocytes, and Myelin Sheaths in Wild‐Type Rapid Myelinating OL Brain Organoids

**FIGURE 1:**
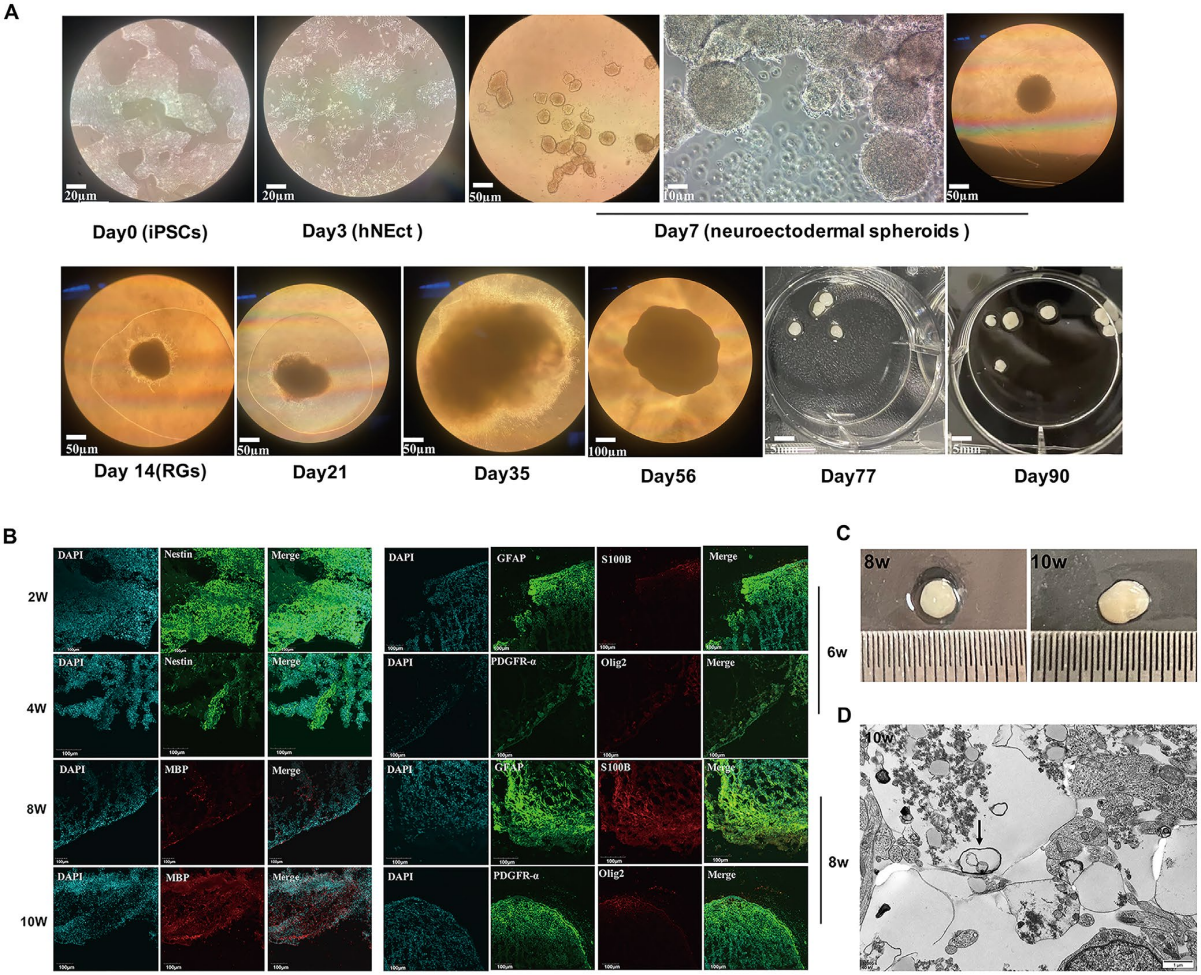
Normal OL brain organoids displayed maturation of astrocytes, oligodendrocytes, and myelin sheaths. (A) Bright‐field images under the microscope of the rapid OL organoids at different days of maturation. (B) Immunofluorescence staining for Nestin (green) and nuclear staining (DAPI, blue) at Week 2 and Week 4. Immunofluorescence staining for GFAP (green), S100B (red), PDGFRα (green), Olig2 (red), and nuclear staining (DAPI, blue) at Week 6 and Week 8. Immunofluorescence staining for MB P (red) at Week 8 and Week 10. Scale bars 100 μm. (C) The diameter of normal organoids at Week 8 and Week 10. (D) Transmission electron microscopy showed the myelin sheath. Scale bars 1 μm.

Following the protocol for generating rapid myelinating human cortical organoids, bright and smooth edges were observed in the neuroectodermal spheroids at approximately Day 7. Subsequently, these spheroids were embedded in Matrigel to facilitate their transformation into OL brain organoids. Over time, in culture, the size of the organoids significantly increased. During the proliferative phase beginning on Day 7, the cerebral organoids showed an expanded epithelium and an increase in size. At Week 2, the radial glial cells (RGs) surrounding the organoids could be observed (Figure 1A). The organoids reached their maximum size of 4.4 mm at approximately Weeks 10–12 (Figure 1C).

The nestin protein is an intermediate silk protein expressed at high levels in cortical radial glia/neural progenitor cells (NPCs). Nestin can be detected in the early stage of brain organoid development. As the organoids proliferate and differentiate, the protein expression of nestin decreases, and nestin may almost disappear. We observed a substantial increase in nestin expression by Week 2 in normal brain organoids, followed by a gradual decrease by Week 4, signifying the differentiation of stem cells to glial cells (Figure 1B).

Subsequent analysis focused on the evaluation of astrocyte activation and maturation through the immunofluorescence of GFAP and S100B, which demonstrated significant increases in their expression levels by Week 6, reaching higher levels by Week 8 in WT brain organoids. Additionally, the expression levels of PDGFRα, a marker of oligodendrocyte progenitor cells (OPCs), and Olig2, a marker of mature oligodendrocytes, were assessed. Our results, as depicted in the figures, indicated marked increases in the numbers of PDGFRα‐positive OPCs and Olig2‐positive OLs at Week 6, with significant increases over time in culture. Moreover, we investigated the expression of MBP, a crucial component of central nervous system myelin, which serves as a marker of myelin function. Our findings revealed the detection of MBP‐positive cells by approximately Week 8, with further observation at Week 10 during the differentiation phase of normal brain organoids (Figure 1B). Notably, electron microscopy of the organoids at Week 10 revealed helical or concentrically coiled myelin‐like structures, indicating successful engagement in myelin deposition (Figure 1D).

In summary, our results revealed notable maturation of astrocytes, oligodendrocytes, and myelin sheaths within 10 weeks, which shortened the cultivation phase by more than half compared to that of the classical brain organoid method described by Lancaster et al. [22]. The rapid myelinating OL brain organoids offer a more efficient model for research in leukodystrophies.

### 3.2 eIF2B Mutation Decreased the Size of OL Cerebral Organoids

**FIGURE 2:**
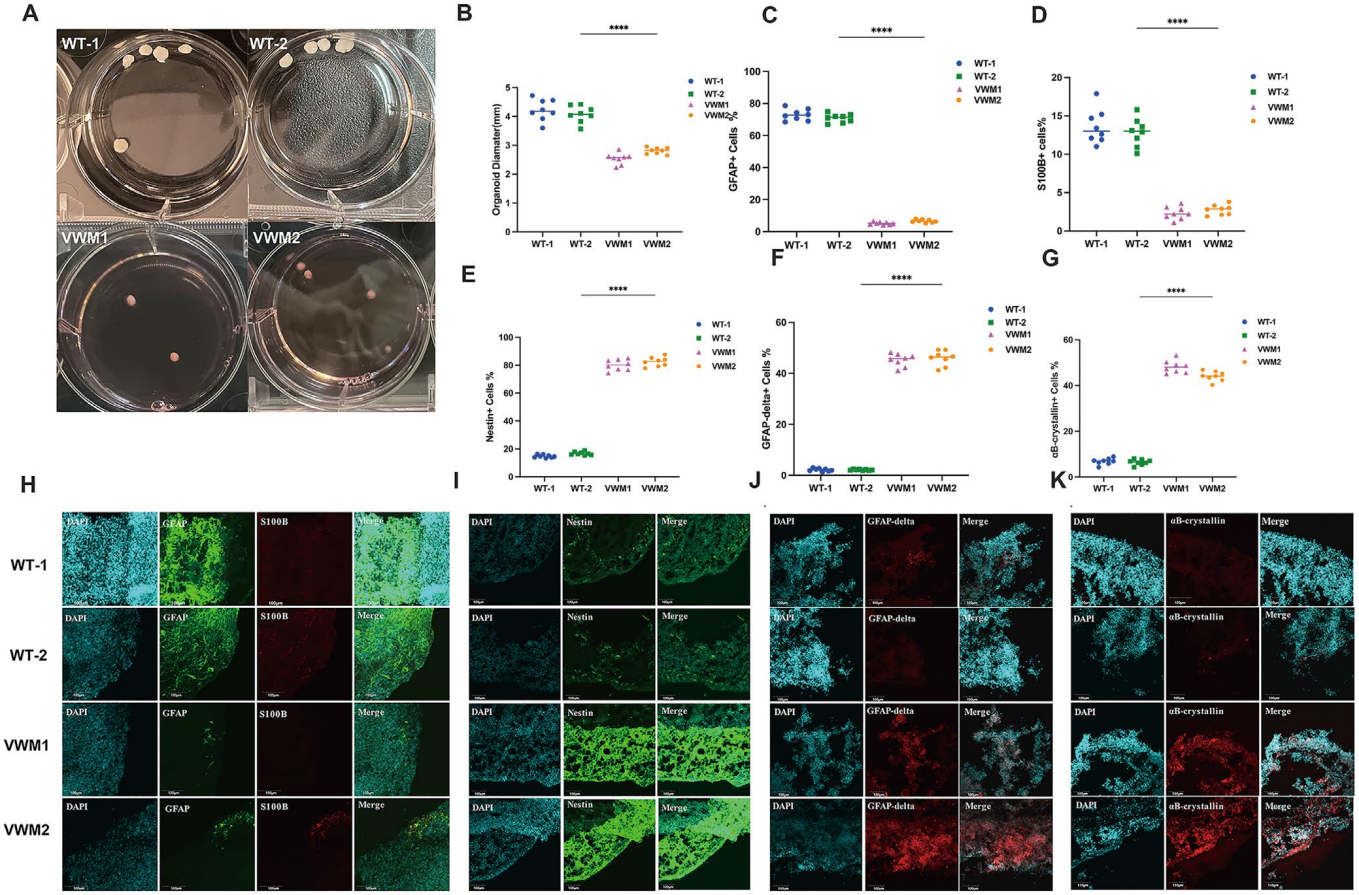
eIF2B mutation led to smaller size and impaired differentiation of astrocytes in cerebral organoids. (A) Bright‐field images of WT and mutant OL cerebral organoids at Week 10. (B) Quantification of the diameter (mm) of the brain organoids. Data were presented as mean ± SEM values, *n* = 8 in each group with three independent experiments; one‐way ANOVA analysis. ns: *p* > 0.05, **p < 0.01, ***p < 0.001. (C–G) Data were presented as mean ± SEM values, *n* = 8 in each group with three independent experiments; one‐way ANOVA analysis and Kruskal–Wallis ANOVA analysis. ns: *p* > 0.05, **p < 0.01, ***p < 0.001. (H) Representative images of immunostaining of the mature astrocyte markers GFAP (green), and S100B (red) and nuclear staining (DAPI, blue) of WT and mutant OL cerebral organoids at Week 8. Scale bars 100 μm. (I) Representative images of immunostaining of marker Nestin (Green) and nuclear staining (DAPI, blue) at Week 6. (J–K) Representative images of immunostaining of the dysfunctional astrocyte markers GFAP‐delta (red), αB‐crystallin (red), and nuclear staining (DAPI, blue) of WT and mutant OL cerebral organoids at Week 8.

To track organoid proliferation and differentiation, we measured organoid diameter during the culture phase. Our results revealed that the mutant OL brain organoids exhibited a developmental delay in comparison to those of the WT group during the same proliferation and differentiation period. The diameters of the mutant cerebral organoids were 26 ± 0.33 mm and 28 ± 0.43 mm, respectively, which were significantly smaller than the 42 ± 0.26 and 44 ± 0.26 mm of the WT at Week 10 (Figure 2A,B).

### 3.3 Increased Numbers of Immature Astrocytes With Decreased GFAP Expression but Increased Expression of Nestin, GFAPδ, and αB‐Crystallin in Mutant Brain Organoids

As shown in Figure 2H,C,D, the proportion of GFAP‐positive astrocytes in mutant OL brain organoids was significantly lower than that in WT organoids, while a lower expression level of the S100B marker was also noted in mutant cerebral organoids at Week 8. Moreover, there was no significant difference between VWM1 and VWM2. GFAP has multiple splice variants. The abnormal isoform GFAPδ has a negative effect on GFAP filament formation. Therefore, GFAPδ can be used as a marker for immature or dysfunctional astrocytes. αB‐Crystallin is a small heat shock protein that is enriched in astrocytes. The results presented in Figure 2F,J demonstrated that the proportion of GFAPδ‐positive astrocytes was significantly greater than that in the WT group. Furthermore, the mutant OL organoids contained more αB‐crystallin‐positive astrocytes than did the WT OL organoids at Week 8 (Figure 2G,K). In addition, the results presented in Figure 2E,I illustrate that the proportion of nestin‐positive cells was significantly greater in the mutant organoids than in the WT organoids at Week 6. These findings indicated that mutations in eIF2B led to astrocyte development delay and dysfunction in 3D organoids.

### 3.4 Mutant OL Brain Organoids Contained Decreased Numbers of OPCs, Mature Oligodendrocytes, and Sparse Myelin

**FIGURE 3:**
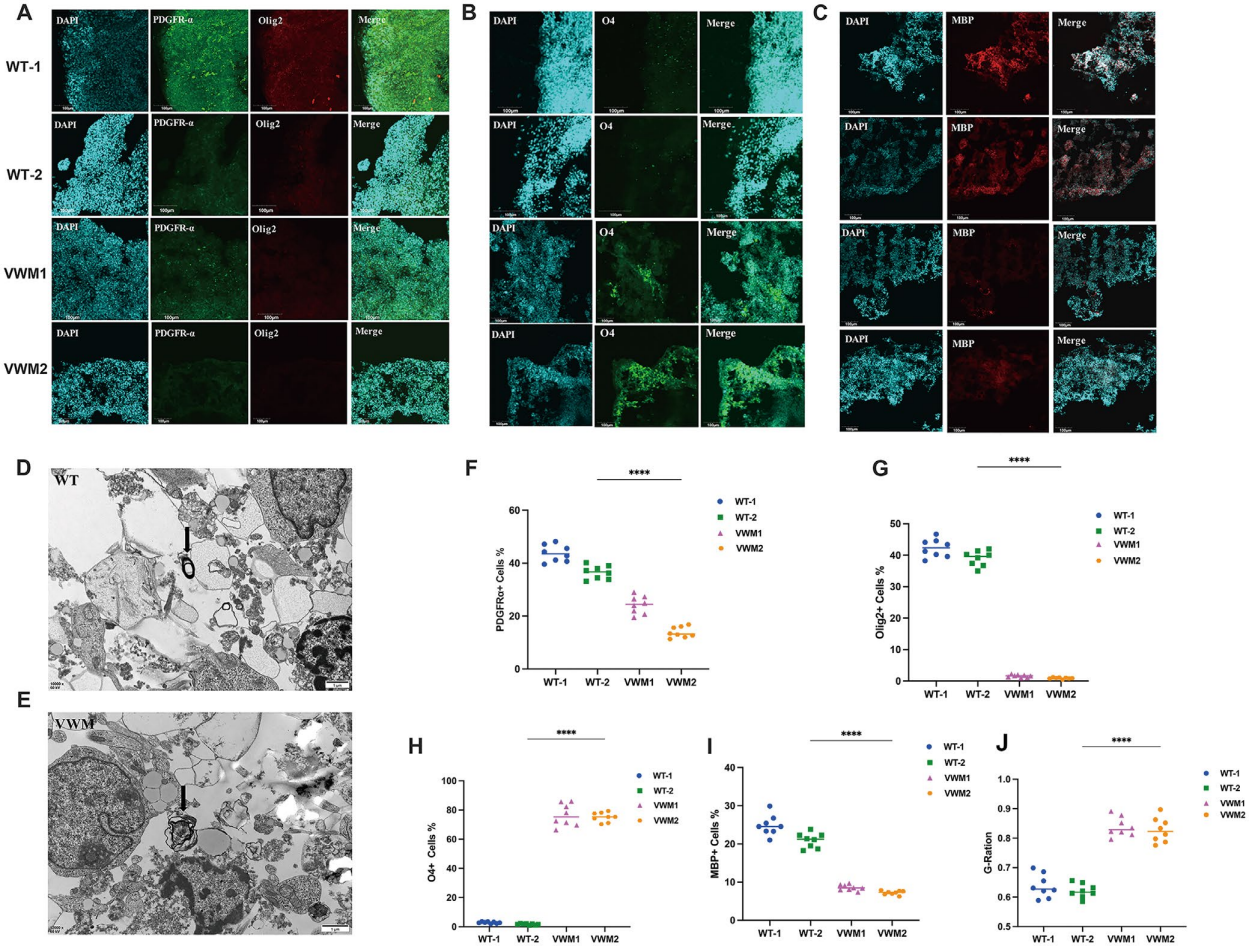
eIF2B mutation resulted in decreased OPCs, mature oligodendrocytes, and sparse myelin. (A) Immunofluorescence staining for OPC marker PDGFRα (green), mature oligodendrocytes marker Olig2 (red), and nuclear staining (DAPI, blue) at Week 8. Scale bars 100 μm. (B) Immunofluorescence staining for immature oligodendrocyte marker O4 (green) and nuclear staining (DAPI, blue) at Week 8. Scale bars 100 μm. (C) Immunofluorescence staining for mature myelin marker MBP (red) and nuclear staining (DAPI, blue) at Week 10. (D,E) Transmission electron microscopy showed helical or concentrically coiled myelin‐like structures in the WT organoids and the sparse myelin sheaths in mutant organoids. Scale bars 1 μm. (F–I) Data were presented as mean ± SEM values, *n* = 8 in each group with three independent experiments; one‐way ANOVA analysis and Kruskal–Wallis ANOVA analysis. ns: *p* > 0.05, **p < 0.01, ***p < 0.001. (J) Quantification of G‐ratio at Week 10 using one‐way ANOVA analysis and Kruskal–Wallis ANOVA analysis. ns: *p* > 0.05, **p < 0.01, ***p < 0.001.

Our experiments revealed notable decreases in the proportions of PDGFRα‐positive OPCs and Olig2‐positive mature oligodendrocytes in mutant brain organoids compared to those in WT organoids at Week 8 (Figure 3A,F,G). In contrast, the level of O4 protein, which is considered a specific marker of immature oligodendrocytes, was significantly greater in the mutant group (Figure 3B,H) at Week 8. Additionally, at approximately Week 10, the MBP protein was highly expressed in WT organoids, and the percentage of MBP‐positive areas in the mutant organoids was lower than that in the WT organoids (Figure 3C,I).

In addition, the electron microscopy results showed marked differences between the WT and mutant organoids. At Week 10, we observed a helical or concentrically coiled myelin‐like structure in the WT organoids, whereas the mutant organoids showed sparse myelin sheaths (Figure 3D,E). The G‐ratio is commonly used to measure the ratio of the axon diameter to the myelinated total fiber diameter and helps to assess the structure and function of the myelin sheath. According to our results (Figure 3J), the G‐ratio was significantly greater in the VWM1 and VWM2 groups than in the control WT group, indicating that the myelin was thinner in the mutant organoids than in the WT organoids.

### 3.5 Hyperactivation of the UPR Signaling Pathway in Mutant OL Brain Organoids

**FIGURE 4:**
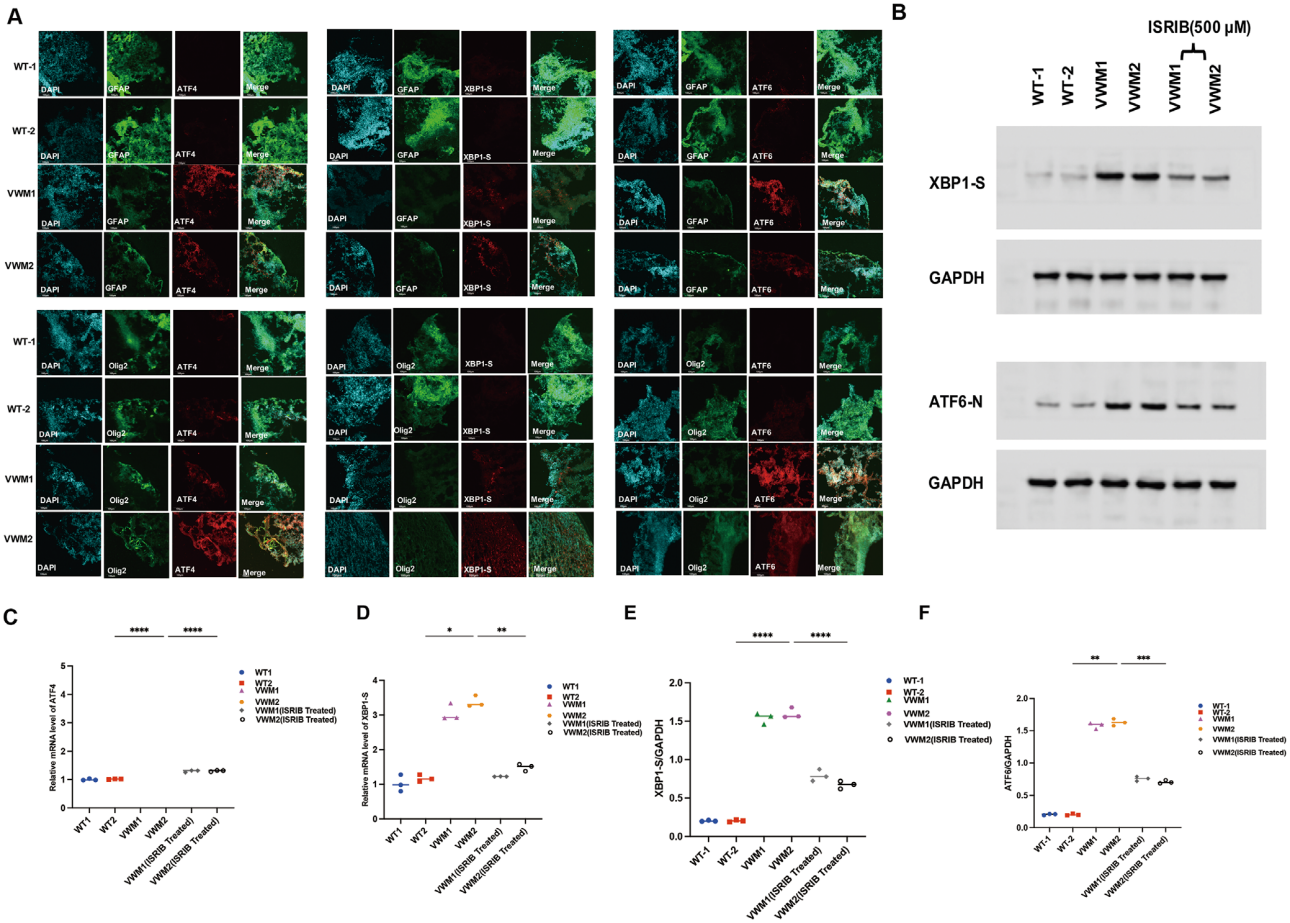
eIF2B mutation led to UPR signaling pathway overactivation and ISRIB treatment alleviated the overactivation of UPR signaling pathway in mutant organoids. (A) Immunofluorescence staining for markers of UPR signaling pathway (ATF4, ATF6, and XBP1‐S, Red), marker of mature astrocytes GFAP (green), maker of mature oligodendrocytes Olig2 (green), and nuclear staining (DAPI, blue) at Week 10. (B) Western blotting of ATF6‐N and XBP1‐S in WT and mutant organoids at Week 8; (C,D) The levels of ATF4 and XBP1‐S in WT and mutant organoids, one‐way ANOVA analysis and Kruskal–Wallis analysis. ns: *p* > 0.05, **p < 0.01, ***p < 0.001. (E,F) Qualification of the levels of XBP1‐S and ATF6‐N in cerebral organoids with three independent experiments at Week 8; Kruskal–Wallis analysis. ns: *p* > 0.05, **p < 0.01, ***p < 0.001.

As shown in Figure 4A, three UPR signaling pathway molecules (ATF4, XBP1‐S, and ATF6) were expressed in mutant brain organoids with WT, which mainly overactivated in GFAP‐positive astrocytes and Olig2‐positive oligodendrocytes. In our study, we also tested the mRNA expression levels of ATF4 and XBP1‐S by q‐PCR. The results showed that the mRNA expression levels of ATF4 and XBP1‐S significantly increased in mutant brain organoids compared to the WT groups (Figure 4C,D). In addition, western blot analysis of brain organoids also revealed the elevated expression levels of ATF6‐N and XBP1‐S proteins. These results all indicated that all 3 arms of the UPR were overactivated in mutant brain organoids in VWM (Figure 4B,E,F).

### 3.6 ISRIB Treatment Increased the Size of Mutant Brain Organoids

**FIGURE 5:**
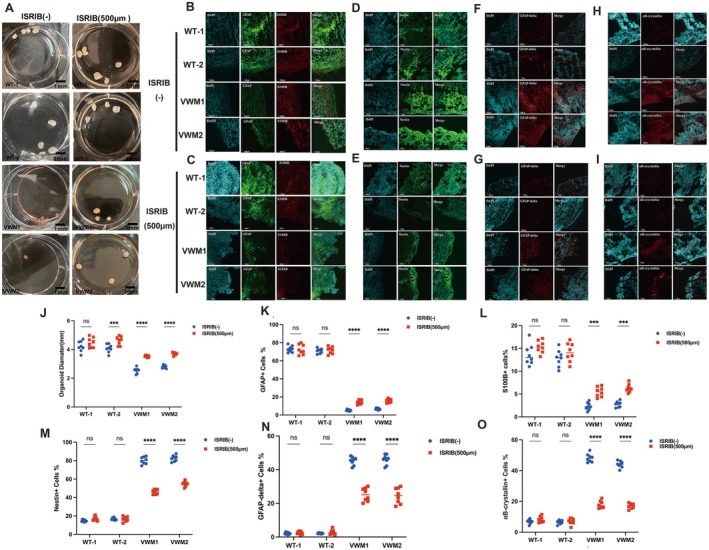
ISRIB treatment contributed to increased size and numbers of mature astrocytes in mutant OL cerebral organoids. (A) Bright‐field images of OL cerebral organoids without ISRIB and treated with ISRIB (500 μm). Scale bars 1 mm. (B,C) Representative images of immunostaining of mature astrocyte markers GFAP (green), and S100B (red) and nuclear staining (DAPI, blue) of WT and mutant OL cerebral organoids without ISRIB and ISRIB treatment (500 μm), respectively, at Week 8. Scale bars 100 μm. (D,E) Representative images of immunostaining of marker nestin (green) and nuclear staining (DAPI, blue) without ISRIB and ISRIB treatment (500 μm), respectively, at Week 6. Scale bars 100 μm. (F–I) Representative images of immunostaining of the dysfunctional astrocyte markers GFAP‐delta (red), αB‐crystallin (red), and nuclear staining (DAPI, blue) of WT and mutant OL cerebral organoids without ISRIB and ISRIB treatment (500 μm), respectively, at Week 8. Scale bars 100 μm. (J) Quantification of the diameter (mm) of the brain organoids after ISRIB treatment (500 μm). Data were presented as mean ± SEM values, *n* = 8 in each group, with three independent experiments; one‐way ANOVA analysis. ns: *p* > 0.05, **p < 0.01, ***p < 0.001. (K–O) Data were presented as mean ± SEM values, *n* = 8 in each group with three independent experiments; one‐way ANOVA analysis and Kruskal–Wallis ANOVA analysis. ns: *p* > 0.05, **p < 0.01, ***p < 0.001.

To investigate the impact of the small‐molecule ISRIB, the WT and mutant organoids were treated with 500 μM ISRIB after the neuroectodermal spheroids were embedded in Matrigel from Week 1. Fresh ISRIB was added when we changed the medium to ensure that the organoids were constantly maintained with ISRIB at an adequate concentration. We found that the diameters of the mutant cerebral organoids were 35 ± 0.43 and 36 ± 0.37 mm at Week 10, indicating that their diameters had significantly increased after ISRIB treatment (Figure 5A,J). These results demonstrated that ISRIB promoted the proliferation of brain organoids.

### 3.7 ISRIB Treatment Contributed to Increased Numbers of Mature Astrocytes, OPCs, Mature Oligodendrocytes, and Myelin in Mutant OL Cerebral Organoids

To evaluate the effects of ISRIB on astrocytes and oligodendrocytes, the typical markers described in Sections 3.3 and 3.4 were also tested in the organoids after ISRIB treatment.

Figure 5H shows that the proportions of GFAP and S100B‐positive astrocytes in the mutant brain organoids were still lower than those in the WT brain organoids after the application of ISRIB. However, the mutant brain organoids contained significantly more GFAP‐positive astrocytes, while the expression level of S100B showed no obvious difference at Week 8 (Figure 5B,C,K,L). Additionally, the expression levels of immature astrocyte markers, including nestin, GFAPδ, and αB‐crystallin, were significantly decreased after ISRIB treatment at Week 8 (Figure 5D–I,L–O). These observations suggest that ISRIB may have positive effects on the proliferation and maturation of astrocytes.

**FIGURE 6:**
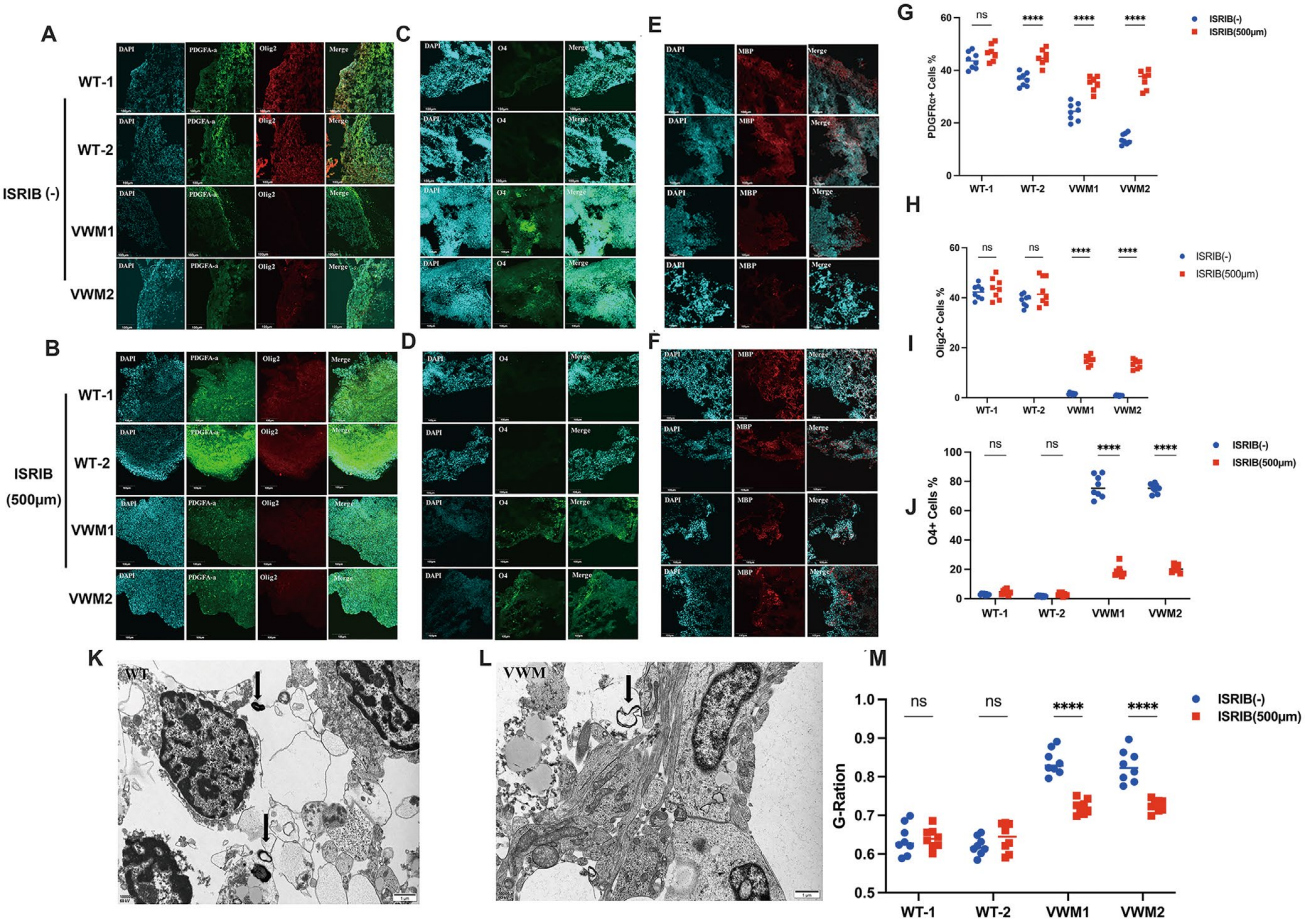
ISRIB treatment increased mature oligodendrocytes and myelin in mutant OL cerebral organoids. (A,B) Immunofluorescence staining for OPC marker PDGFRα (green) and mature oligodendrocytes marker Olig2 (red) and nuclear staining (DAPI, blue) without ISRIB and ISRIB treatment (500 μm), respectively, at Week 8. Scale bars 100 μm. (C,D) Immunofluorescence staining for mature oligodendrocyte marker O4 (green) and nuclear staining (DAPI, blue), without ISRIB and ISRIB treatment (500 μm), respectively, at Week 8. Scale bars 100 μm. (E,F) Immunofluorescence staining for mature myelin marker MBP (red) and nuclear staining (DAPI, blue) without ISRIB and ISRIB treatment (500 μm), respectively, at Week 10. (G,H) Transmission electron microscopy showed the helical myelin‐like structure in the WT and mutant organoids after ISRIB treatment (500 μm) at Week 10. Scale bars 1 μm. (I–L) Data were presented as mean ± SEM values, *n* = 8 in each group with three independent experiments; one‐way ANOVA analysis and Kruskal–Wallis ANOVA analysis. ns: *p* > 0.05, **p < 0.01, ***p < 0.001. (M) Quantification of G‐ratio after ISRIB treatment at Week 10; one‐way ANOVA analysis and Kruskal–Wallis ANOVA analysis. ns: *p* > 0.05, **p < 0.01, ***: *p* < 0.001.

Similarly, the proportions of PDGFRα‐positive OPCs and Olig2‐positive oligodendrocytes in mutant brain organoids were markedly increased after ISRIB application, while there was a significant decrease in the expression of the O4 protein at Week 8 (Figure 6A–D,I–K). At Week 10, the expression level of MBP increased in the mutant cerebral organoids compared with that before ISRIB treatment (Figure 6E,F,L). Electron microscopy revealed similar results, with more helical or concentrically coiled myelin‐like structures observed (Figure 6G,H). In addition, the G‐ratio of mutant organoids calculated by ImageJ decreased significantly after ISRIB treatment, indicating an improvement in the development of myelin sheaths due to ISRIB (Figure 6M).

### 3.8 ISRIB Treatment Inhibited the Overactivation of UPR Pathway in Mutant Organoids

The crosstalk between the ISR and the UPR led us to hypothesize that ISRIB would exert a dominant effect on regulating the UPR pathway. As illustrated in Figure 6, the mRNA expression levels of XBP1‐S and ATF4 were markedly reduced following treatment with ISRIB, consistent with our prediction (Figure 4C,D). In addition, the western blot results showed the protein levels of XBP1‐S and ATF6‐N of eIF2B mutant brain organoids decreased significantly after ISRIB treatment (Figure 4B,E,F). Cumulatively, the evidence suggests that ISRIB functions as an inhibitor of the UPR signaling pathway.

## 4 Discussion

Recently, in the field of neuroscience, particularly in studies of neurodegenerative disorders, three‐dimensional brain organoid technology has come to be considered a crucial tool for understanding pathological mechanisms and exploring new therapeutic approaches in vitro [22, 23]. Brain organoids are typically induced from human pluripotent stem cells, such as T Cells from human peripheral blood or human dermal fibroblast‐derived iPSCs, and have demonstrated the ability to better replicate genetic characteristics in vitro [23, 24]. Moreover, the development of diverse region‐specific brain organoid protocols has facilitated the generation of specialized cells or structures, offering more effective and selective strategies for studying various neurological disorders [25, 26]. Compared with 2D cell or animal models, 3D brain organoids contain a diverse range of neuronal cell lines and have tighter cell–cell interactions, making them superior models for simulating neuronal cell growth, differentiation, or self‐organization and replicating the phenotypic and genetic characteristics of associated diseases. The first eIF2B mutant brain organoid model from iPSCs from a patient with VWM was established by Deng et al. [19]. and was shown to mimic the dynamic brain development process, particularly early brain organization, similar to fetal brain development. According to their organoid method, GFAP‐positive astrocytes were observed at nearly Week 12 in normal organoids, while they appeared in mutant organoids after 100 days of culture. Additionally, Olig‐2‐positive oligodendrocytes appeared at Week 20 in the mutant organoids, similar to those in the WT organoids. Furthermore, the detection of mature myelin sheaths took more than 180 days in the WT organoids and even longer in the eIF2B mutant organoids. Although this method has the advantages of modeling human brain development and simulating the disease process of VWM, it has several limitations. For instance, the culture period of the brain organoids in this protocol was no less than 12 weeks for the observation of critical glial cells responsible for VWM and even longer for the detection of MBP. The absence of the vascular system, immune cells, nutrients, and cerebrospinal fluid flow in brain organoids leads to hypoxia, increased cellular stress, and an altered brain environment, resulting in increased cell apoptosis and necrosis during long‐term cultivation. Additionally, long‐term culturing of organoids is associated with an increased risk of cellular contamination [27, 28]. To address the limited availability of classical human brain organoid methods, we established a cerebral organoid model for VWM disease using an accelerated brain development process based on Mohammed R. Shaker's method [20]. The use of growth factors such as HGF, NT3, and T3 in the early stage (i.e., during the formation of 3D neuroectodermal spheroids) plays a critical role in promoting the maturation of oligodendrocytes and functional myelination.

In our experiments, mature oligodendrocytes were initially observed at Week 6 and were highly abundant at Weeks 8–9, along with mature astrocytes. These results are in contrast to the organoid protocol of VWM constructed by Deng et al., in which mature oligodendrocytes appeared later, indicating the acceleration of the maturation process in our method. The early appearance of mature oligodendrocytes and GFAP‐positive astrocytes in our organoid model aligns with the findings of Mohammed R. Shaker. Moreover, markers of mature oligodendrocytes Olig2‐ and GFAP‐positive astrocytes were observed in brain organoids constructed by Mohammed R. Shaker from an even earlier stage at Week 3. The quality of neuroectodermal spheroids and the stability of the cell culture environment significantly influence the brain development process in organoids, thus allowing reasonable differences in the organoid development process during practical cultivation. In addition, our observations of the sheath‐like morphology of MBP‐encompassing axons at Week 10 indicated successful and early myelination in our organoid model. Notably, the occurrence timings of different markers associated with critical component cells in the organoids were similar between the WT and mutant groups in our experiments. We speculated that the greatly shortened duration of organoid cultivation led to the rapid proliferation and differentiation of glial cells in vitro; thus, the WT and mutant organoids expressed representative protein markers at similar time points. However, there were significant differences in the expression levels of representative protein markers between the WT and mutant organoids. Another notable finding in our study was that the proportions of PDGFRα‐positive oligodendrocyte precursor cells (OPCs) and Olig2‐positive oligodendrocytes in the mutant organoids were both lower than those in the wild‐type (WT) organoids. A previous study by Deng et al. also reported a lower number of Olig2‐positive oligodendrocytes in the mutant organoids compared to the WT organoids. However, they found that the number of PDGFRα‐positive OPCs was significantly higher in the mutant organoids than in the WT organoids. This suggests that the delay in oligodendrocyte development in the mutant organoids led to the accumulation of OPCs. In contrast, the presence of a large number of OPCs alongside mature oligodendrocytes in the normal organoids indicates more efficient proliferation and differentiation of oligodendrocytes during the rapid and shortened differentiation process observed in the organoids. In addition, the electron microscopy results in our study revealed the accelerated deposition of myelin wrapping around axons, which provided clearer and solid evidence for successful myelination in this rapid organoid method.

According to past research aimed at the natural history of VWM, 60% of patients had symptoms before the age of 4 years, and the age of onset is a critical determinant of prognosis. VWM patients with onset at < 1 year have rapid disease progression and usually die within several months, while patients with onset at 1 to < 2 years often show motor decline after months to a few years and die within several years [29, 30]. Consequently, the need for effective and safe treatments beyond symptomatic care has become increasingly apparent. Previous studies have indicated that the deregulation of the ISR plays a central role in VWM pathology, suggesting that the ISR could serve as a promising drug target for treating VWM. The drug‐like compound, ISRIB, has shown promise in ameliorating abnormal ISR activation [31, 32]. Previous studies have demonstrated that ISRIB enhances the GEF activity of eIF2B in the presence of eIF2 and attenuates ATF4‐mediated gene expression, thereby alleviating translational inhibition triggered by abnormal ISR activation [33]. Moreover, ISRIB has exhibited beneficial effects in various models of neurocognitive disorders, dysmyelination, inflammation, head injury, and cancer [34, 35, 36]. For instance, ISRIB could improve cognitive function and memory in normal mice and in the mice after brain injury [35]. In the model of several neuropsychiatric disorders, ISRIB has been shown to alleviate the social deficit and elevated anxiety‐like behavior [37]. In the context of VWM, studies have shown that the expression of ATF4 and the ATF4‐regulated transcriptome, induced by reduced eIF2B activity, was increased in mice with biallelic missense mutations in eIF2B, as well as in brain tissues from VWM patients. Administration of ISRIB and analogs to VWM mice normalized the expression of specific mRNA markers altered by eIF2B mutations, ameliorated brain white matter pathology, and improved motor skills [13, 15, 16]. Recently, the ISRIB‐like molecule DNL343 is currently being evaluated in late‐stage clinical trials in people living with amyotrophic lateral sclerosis (ALS) based on its ability to improve cellular homeostasis and prevent neuronal degeneration [16, 38]. Another ISRIB analog Fosigotifator (ABBV‐CLS‐7262) is being evaluated for safety, tolerability, and pharmacokinetics in participants diagnosed with VWM disease. This study is the first time an eIF2B activator has been administered to people with VWM disease (NCT05757141).

Our study represents the first investigation of the effects of ISRIB on brain organoids. We observed a pronounced and direct inhibitory effect of ISRIB on the UPR signaling pathway and significant improvements in the maturation of astrocytes, oligodendrocytes, and myelin sheaths in mutant organoids. Our findings provide compelling evidence of the ability of ISRIB to enhance the activity and maturation of glial cells in eIF2B‐mutant organoids, thereby supporting its potential as a novel drug for VWM. While ISRIB has shown promising benefits in models of neurodegenerative and dysmyelination disorders, further clinical trials or other investigations are warranted to evaluate its safety and tolerability. Concerns have been raised regarding the potential long‐term risk of cancer associated with ISRIB, given its ability to enhance eIF2B activity and stimulate growth, as suggested by previous studies.

Although this rapid protocol for human cortical organoid cultivation offers significant advantages in shortening the cultivation phase, it also has certain limitations. First, we observed significant variation in the size of different batches of organoids following this protocol, indicating a need to improve batch reliability. Only two cases of VWM patient‐derived iPSCs and two normal control iPSCs were used in our experiment; the small sample size may reduce the representability of research results to some extent. Second, in our experiments, the organoids were cultured for up to 16 weeks, during which severe apoptosis occurred, leading to volume shrinkage and necrosis of the hollow portion of the brain organoids. Therefore, it is crucial to develop methods to maintain these organoids for longer periods. Additionally, due to the rapid maturation process, this method is not a comprehensive and accurate model for observing dynamic brain development during different phases. The time points selected in our study to observe the brain development phase are limited to fully reproduce the development of the nervous system. In addition, the absence of myelination by observing the formation and structure of the myelin sheath was also found in the study. Despite our findings suggesting the activation of all three branches of the unfolded protein response (UPR) pathways, the interpretation of these results remains contentious. The observed overactivation may be attributed to an artifact arising from the rapid differentiation protocol employed in vitro, wherein cells are subjected to metabolic stress or hypoxic conditions, potentially leading to confounding results. Further investigations into the UPR pathway during the early stage of VWM disease are essential for a comprehensive understanding of its pathophysiology.

## 5 Conclusions

In conclusion, we constructed a rapid and efficient brain organoid model of VWM for the first time that generated mature astrocytes, oligodendrocytes, and myelin sheaths within 10 weeks in vitro. To the best of our knowledge, this rapid organoid protocol represents the fastest method to date for generating mature oligodendrocytes and myelin sheaths in human cerebral cortical organoids. These findings provide more evidence that this rapid brain organoid method is a reliable and more effective tool for investigating the underlying pathological mechanisms of VWM and other CNS degenerative disorders. Further research involving coculture with essential cell types, such as vascular or immune cells, could enhance the ability of the model to mimic human brain development and improve neuronal survival. Additionally, our results indicated that ISRIB has the ability to alleviate overactivation of the UPR signaling pathway and enhance the maturation of glial cells and myelin sheaths in organoids, suggesting its potential as a candidate therapeutic for VWM and other neurological disorders characterized by abnormal ISR activation.

## Author Contributions

W.Y., J.D., and Y.W. designed the study. W.Y., J.D., K.G., and H.Y. performed the experiments and analyzed the data. W.Y. and J.D. wrote the manuscript. W.Y., J.D., J.Z., F.Z., J.W., Y.W., and Y.W. participated in data analysis and data interpretation. Y.W. and Y.J. conceived and supervised the study. All authors contributed to the discussion and revision of the manuscript.

## Conflicts of Interest

The authors declare no conflicts of interest.

## Supporting information


Figure S1.

**Figure S2.** The full unedited blots for Figure 4. The blots contained markers denoting the location of molecular weight standards (XBP1‐S 56KD; ATF6‐N 50KD; GAPDH 37KD). The western blot experiments were performed three times.

**Figure S3.** The full unedited blot for ATF6‐full length and ATF6‐N. The blot contained markers denoting the location of molecular weight standards (ATF6‐N 50KD; ATF6 full‐length 90KD; GAPDH 37KD). The full‐length ATF6 and the active‐form ATF6‐N were both detected by the antibody ab122897 from Abcam.

## Data Availability

The data that support the findings of this study are available from the corresponding author upon reasonable request.
