# Supplementary figures and images for: Effects of the Small‐Molecule ISRIB on the Rapid and Efficient Myelination of Oligodendrocytes in Human Stem Cell‐Derived Cerebral Organoids in Patients With Leukoencephalopathy With Vanishing White Matter
Source: CNS Neurosci Ther. 2025 Apr 28;31(4):e70398. doi: 10.1111/cns.70398 (PMC12037694; doi:10.1111/cns.70398)

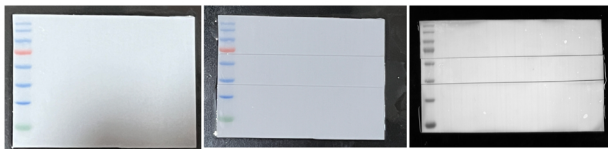

XBP1-S(56KD)

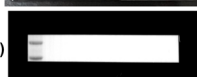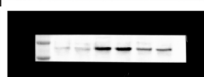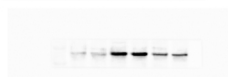

GAPDH(36KD)

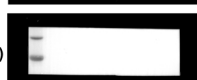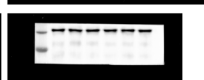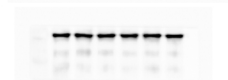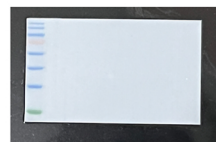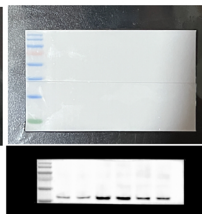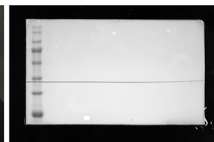

ATF6-N(50KD)

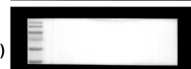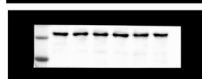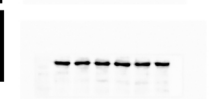

GAPDH(36KD)

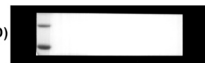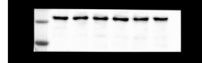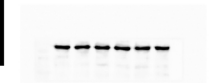

Supplement: Supplementary file 1 — Figure S1. [file CNS-31-e70398-s002.pdf]

XBP1-S(56KD)

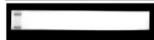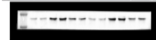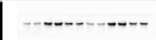

GAPDH(36KD)

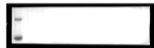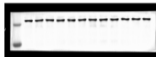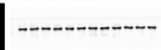

ATF6-N(50KD)

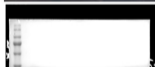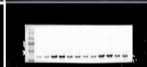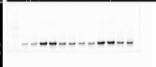

GAPDH(36KD)

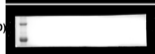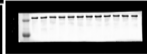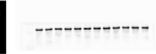

Supplement: Supplementary file 2 — Figure S2. The full unedited blots for Figure 4. The blots contained markers denoting the location of molecular weight standards (XBP1‐S 56KD; ATF6‐N 50KD; GAPDH 37KD). The western blot experiments were performed three times. [file CNS-31-e70398-s003.pdf]

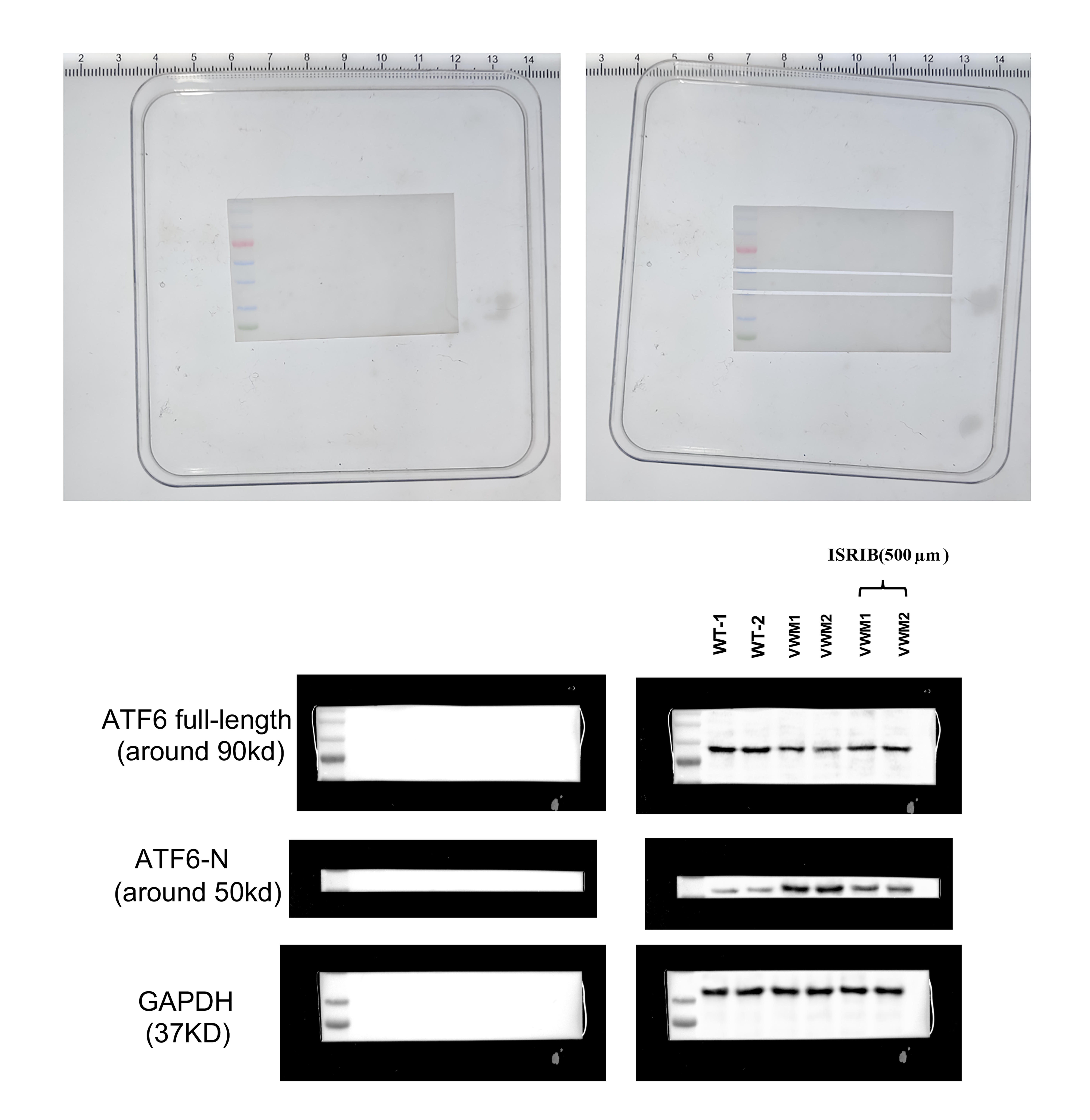

Supplement: Supplementary file 3 — Figure S3. The full unedited blot for ATF6‐full length and ATF6‐N. The blot contained markers denoting the location of molecular weight standards (ATF6‐N 50KD; ATF6 full‐length 90KD; GAPDH 37KD). The full‐length ATF6 and the active‐form ATF6‐N were both detected by the antibody ab122897 from Abcam. [file CNS-31-e70398-s001.tif]
